# Control of *Francisella tularensis* Intracellular Growth by Pulmonary Epithelial Cells

**DOI:** 10.1371/journal.pone.0138565

**Published:** 2015-09-17

**Authors:** Savannah Maggio, Kazuyo Takeda, Felicity Stark, Anda I. Meierovics, Idalia Yabe, Siobhan C. Cowley

**Affiliations:** 1 Laboratory of Mucosal Pathogens and Cellular Immunology, Division of Bacterial, Parasitic and Allergenic Products, Center for Biologics Evaluation and Research, U.S. Food and Drug Administration, Silver Spring, Maryland, United States of America; 2 Microscopy and Imaging Core Facility, Division of Bacterial, Parasitic and Allergenic Products, Center for Biologics Evaluation and Research, U.S. Food and Drug Administration, Silver Spring, Maryland, United States of America; 3 National Research Council Canada, Human Health Therapeutics Portfolio, Ontario, Canada; 4 National Heart, Lung, and Blood Institute, National Institutes of Health, Bethesda, Maryland, United States of America; Albany Medical College, UNITED STATES

## Abstract

The virulence of *F*. *tularensis* is often associated with its ability to grow in macrophages, although recent studies show that *Francisella* proliferates in multiple host cell types, including pulmonary epithelial cells. Thus far little is known about the requirements for killing of *F*. *tularensis* in the non-macrophage host cell types that support replication of this organism. Here we sought to address this question through the use of a murine lung epithelial cell line (TC-1 cells). Our data show that combinations of the cytokines IFN-γ, TNF, and IL-17A activated murine pulmonary epithelial cells to inhibit the intracellular growth of the *F*. *tularensis* Live Vaccine Strain (LVS) and the highly virulent *F*. *tularensis* Schu S4 strain. Although paired combinations of IFN-γ, TNF, and IL-17A all significantly controlled LVS growth, simultaneous treatment with all three cytokines had the greatest effect on LVS growth inhibition. In contrast, Schu S4 was more resistant to cytokine-induced growth effects, exhibiting significant growth inhibition only in response to all three cytokines. Since one of the main antimicrobial mechanisms of activated macrophages is the release of reactive nitrogen intermediates (RNI) via the activity of iNOS, we investigated the role of RNI and iNOS in *Francisella* growth control by pulmonary epithelial cells. NOS2 gene expression was significantly up-regulated in infected, cytokine-treated pulmonary epithelial cells in a manner that correlated with LVS and Schu S4 growth control. Treatment of LVS-infected cells with an iNOS inhibitor significantly reversed LVS killing in cytokine-treated cultures. Further, we found that mouse pulmonary epithelial cells produced iNOS during *in vivo* respiratory LVS infection. Overall, these data demonstrate that lung epithelial cells produce iNOS both *in vitro* and *in vivo*, and can inhibit *Francisella* intracellular growth via reactive nitrogen intermediates.

## Introduction

*Francisella tularensis* is a zoonotic facultative intracellular bacterium that causes a lethal febrile illness in humans known as tularemia. *F*. *tularensis* can infect the host via multiple routes, including the respiratory and gastrointestinal tracts, as well as through broken skin. Respiratory tularemia is the most lethal form of the disease; inhalation of as few as 10 CFU of the highly virulent *F*. *tularensis* subspecies *tularensis* (*F*.*t*. *tularensis*) is sufficient to establish infection, and the mortality rate of untreated pneumonic tularemia is estimated to be as high as 60% [1]. For these reasons, virulent *F*.*t*. *tularensis* was developed as a biological weapon in the mid-20^th^ century, and remains a high priority agent identified as a risk to national security by the United States Centers for Disease Control.

The attenuated live vaccine strain (LVS) of *F*. *tularensis* was derived by repeated passage of a virulent *F*. *tularensis* subspecies *holarctica* strain on agar; LVS has been studied as an investigational product but is not currently licensed for use in humans in the United States [2]. Thus far the protective efficacy of LVS and the key mechanisms of immunity to tularemia remain only partially characterized. In order to better understand the LVS vaccine, and to facilitate the development of new vaccines and therapies against highly lethal pneumonic tularemia, it is important to identify the immune mechanisms that limit respiratory *F*. *tularensis* infection. As an additional benefit, discoveries defining immunity to pulmonary *F*. *tularensis* infection may be applied to other respiratory intracellular pathogens, such as *Mycobacterium tuberculosis*.

*F*. *tularensis* has been detected within alveolar macrophages and airway dendritic cells within one hour after murine pulmonary infection, although the bacteria quickly invade a myriad of other cell types, including lung monocytes, neutrophils, and alveolar type II epithelial (ATII) cells [3]. The majority of these cell types are professional phagocytes that produce multiple anti-microbial factors, such as degradative enzymes, reactive oxygen and nitrogen intermediates, and cationic peptides to inhibit pathogen growth. In particular, macrophages are well known to become activated by interferon-gamma (IFN-γ) and tumor necrosis factor alpha (TNF) to produce reactive nitrogen intermediates (RNI) through induction of the enzyme inducible nitric oxide synthase (iNOS) [4–7]. iNOS produces nitric oxide (NO), which together with other oxidative products such as peroxynitrite and S-nitrosothiols, exert microbiocidal activities [8]. The importance of iNOS to immune defense is reflected by the fact that iNOS-deficient mice are susceptible to sublethal LVS infections [9], and chemical inhibition of iNOS activity significantly inhibits IFN-γ-induced killing of LVS and virulent *F*. *tularensis* in peritoneal exudate macrophages *in vitro* [10, 11]. Overall, macrophage-derived nitric oxide production is considered an important mechanism by which macrophages kill intracellular pathogens, including *Mycobacterium tuberculosis*, *Salmonella typhimurium*, and *Leishmania donovani*.

In contrast to macrophages, ATII cells are not professional phagocytes, although they readily support *Francisella* growth both *in vitro* and *in vivo* [3, 12]. Since ATII cells comprise 15% of all lung cells [13], they have the potential to provide a significant cellular niche for *Francisella* replication during pulmonary infection. Importantly, a Δ*pyrF Francisella* mutant that grew poorly in macrophages but vigorously in other cell types retained full virulence in the murine pulmonary infection model, demonstrating that growth in non-macrophage cell types significantly contributes to *Francisella* virulence [14]. Despite the fact that pulmonary epithelial cells are a potentially unique replication site for *Francisella* in the lungs, little is known about their capacity to inhibit *Francisella* intracellular growth.

Since the immune mechanisms involved in control of *F*. *tularensis* growth in pulmonary epithelial cells will likely provide insights into defense against respiratory *Francisella* infection, here we sought to investigate the possibility that cytokines can activate these cells to produce anti-microbial factors that inhibit *Francisella* growth. Indeed, we show that combinations of the cytokines IFN-γ, TNF, and IL-17A activate murine pulmonary epithelial cells to inhibit the intracellular growth of LVS and the highly virulent *F*. *tularensis* Schu S4 strain. Gene expression analyses revealed up-regulation of NOS2 in *F*. *tularensis*-infected, cytokine-treated cells in a manner that correlated with *Francisella* growth control. Treatment of LVS-infected pulmonary epithelial cells with an iNOS inhibitor significantly reversed LVS killing in cytokine-treated cultures, establishing iNOS as a major antimicrobial mechanism used by these cells to inhibit bacterial intracellular growth. Further, mouse lung ATII cells produced iNOS following *in vivo* sublethal LVS intranasal infection. Collectively, the data presented here demonstrate that lung epithelial cells actively produce iNOS both *in vitro* and *in vivo*, and that these cells possess the capacity to restrict *Francisella* intracellular growth via reactive nitrogen intermediates.

## Materials and Methods

### Bacteria

*F*. *tularensis* LVS (ATCC 29684) was obtained from the American Type Culture Collection. *F*. *tularensis* Schu S4 was obtained from the *Francisella* Strain Collection, Swedish Defense Research Agency, Sweden. Viable bacteria were quantified by plating serial dilutions on supplemented Mueller-Hinton agar (MHA) plates.

### TC-1 cells

The TC-1 cell line was purchased from ATCC, and can now be obtained from Johns Hopkins Technology Ventures. TC-1 cells are pulmonary epithelial cells derived from primary lung cells of C57BL/6 mice cotransformed with HPV-16 E6 and E7 and c-Ha-*ras* oncogenes [15].

### *In vitro* assessment of control of intracellular bacterial growth in TC-1 cells

TC-1 cells were cultured in Dulbecco minimal essential medium (DMEM; Life Technologies) supplemented with 10% heat-inactivated fetal calf serum (FCS; HyClone), 1 mM HEPES buffer (Life Technologies), and 0.1 mM nonessential amino acids (Life Technologies) (complete DMEM; cDMEM), and plated at 2 x 10^5^ viable cells per well in 24-well plates. After overnight culture, TC-1 cells were infected with *F*. *tularensis* LVS or SchuS4 at a multiplicity of infection (MOI) of 1:1 (bacterium-to-TC-1 cell ratio) for 2 hours, washed with PBS, incubated with 50 μg/ml gentamicin for 45 minutes, washed twice with warm PBS, and cDMEM with or without recombinant cytokines was added to the wells, as indicated. All cytokines (IFN-γ, TNF, IL-17A, and IL-22) were obtained from BioLegend, and used in experiments at a concentration of 100 ng/ml, with the exception of TNF, which was used at a concentration of 50 ng/ml. Cultures were incubated at 37°C in 5% CO_2_ for the remainder of the experiment. Bacterial uptake or recovery was determined after initial infection, and after 72 hours of culture, by washing the monolayers once with warm PBS, followed by lysis of infected cells with sterile distilled water, plating on agar, and counting. Supernatants were obtained from the cultures after 72 hours for nitrite analyses.

### RNA extraction and quantitative real time RT-PCR

Total RNA was prepared using RNeasy Mini Kit (Qiagen), and first-strand cDNA was synthesized using the Retroscript Kit (Ambion) according to the manufacturer's instructions. Real time PCR was performed using the ABI Prism ViiA7 instrument (Applied Biosystems). NOS2 transcripts were quantified using TaqMan^®^ Pre-developed Assay Kits (Applied Biosystems) according to the manufacturer's instructions, and the results normalized to expression of GAPDH. The relative standard curve method was used to derive normalized expression for all samples, and “NOS2 fold induction” was calculated as the ratio of cytokine-treated samples to “LVS alone” or “Schu S4 alone” control samples.

### Quantitation of RNI in alveolar epithelial cell culture supernatants

Culture supernatants were assayed for nitrite by the Griess reaction using a commercial Griess reagent according to the manufacturer's instructions (Sigma).

### *In vivo* mouse infections

Male specific pathogen free C57BL/6J mice were purchased from Jackson Laboratory. Animals were housed in a barrier environment at CBER/FDA, and the FDA Institutional Animal Care and Use Committee approved this study. Intranasal infections were performed by delivering 2 x 10^2^ LVS CFUs in a volume of 25 μl per nare to anesthetized mice. Bacteria for infection were diluted in PBS (Cambrex) containing <0.01 ng/ml endotoxin.

### Immunohistochemistry

Six C57BL/6 strain mice were intranasally infected with 2 x 10^2^ CFU of LVS and six uninfected mice were used as controls (naïve). Seven days after infection, lungs were harvested from the mice. Mouse lungs were inflated with 4% paraformaldehyde installed via the trachea, and fixed for 24 hours at room temperature. The paraffin sections were made by standard procedure for immunofluorescence staining. A dual immunofluorescence staining method was performed to demonstrate immunoreactivity for a combination of mouse monoclonal and rabbit polyclonal antibodies against iNOS and pro-Surfactant protein C (ab129372, Abcam, and Ab3786, Millipore, respectively). Briefly, the sections were deparaffinized, rehydrated and then incubated with 5% normal donkey serum for 30 min at room temperature. Sections were subsequently incubated with primary antibodies against iNOS and pro-SP-C overnight at 4°C. The sections were then incubated with secondary antibodies conjugated with Alexa fluor488 and Alexa fluor594 (Jackson Immunoresearch Laboratories). Hoechst 33258 (Life Technologies) was used for counterstaining at 0.5ug/ml concentration. Fluorescence microscopic images were obtained using a Leica TCS_SP8 DMI6000 confocal microscope system (Leica Microsystems). Images were acquired at 63 x Objective lens (N.A. 1.4) for Alexa 488 and 594 emission wavelengths and stored as lif format for further analyses.

### Statistical Analyses

**Fig 1:**
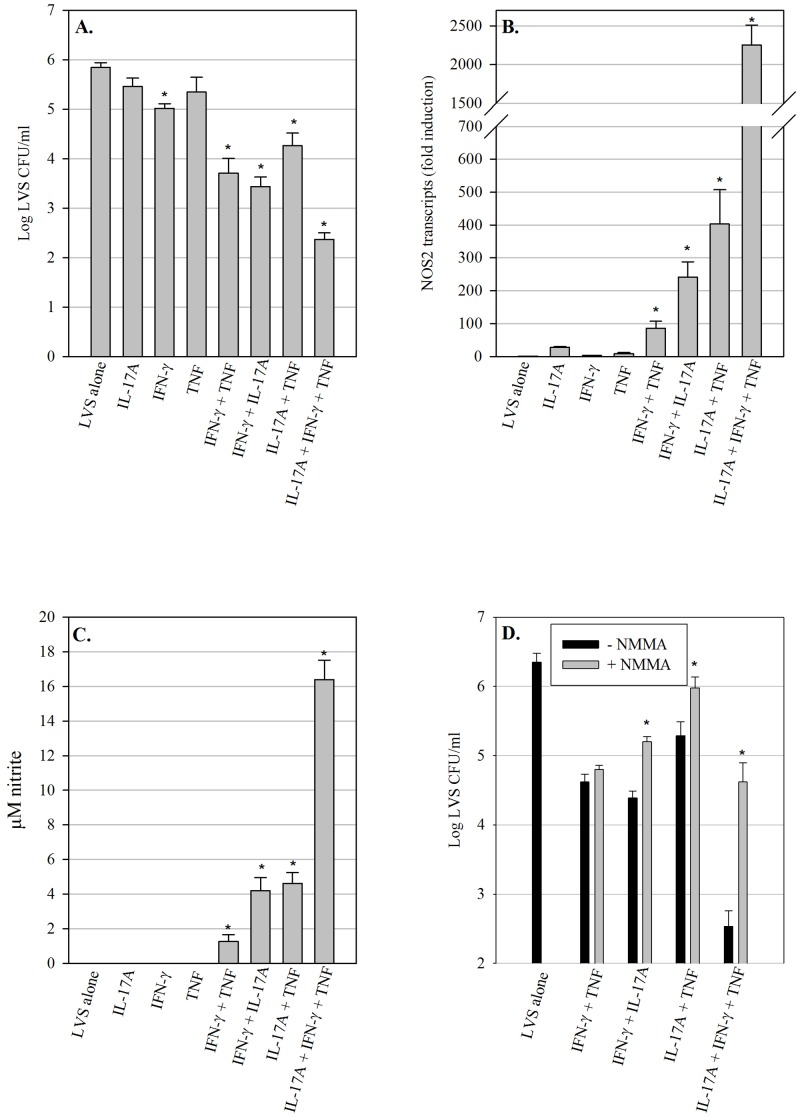
Cytokine-induced control of *F*. *tularensis* LVS growth in pulmonary epithelial cells is partially dependent upon RNI. (A) TC-1 lung epithelial cells were infected with LVS, and intracellular bacteria were enumerated after 72 h. Cultures were treated with recombinant cytokines immediately after infection as indicated, or untreated (‘LVS alone’). * = significantly different from ‘LVS alone’ cultures (P<0.05). (B) Changes in expression of NOS2 in TC-1 cell cultures infected with LVS and treated with recombinant cytokines. Fold induction values are expressed relative to ‘LVS alone’ cultures. * = significantly different from cultures treated only with single cytokines (P<0.05). (C) Nitrite production in TC-1 cell cultures infected with LVS and treated with recombinant cytokines after 72 h. * = significantly different from ‘LVS alone’ cultures (P<0.05). (D) 72 h growth of LVS in cytokine-treated TC-1 cultures in the presence of NMMA. * = significantly different from LVS growth in identically treated cultures in the absence of NMMA (P<0.05). Data are expressed as the mean ± SD of triplicate cultures, and plots are representative of three experiments of similar design.

**Fig 2:**
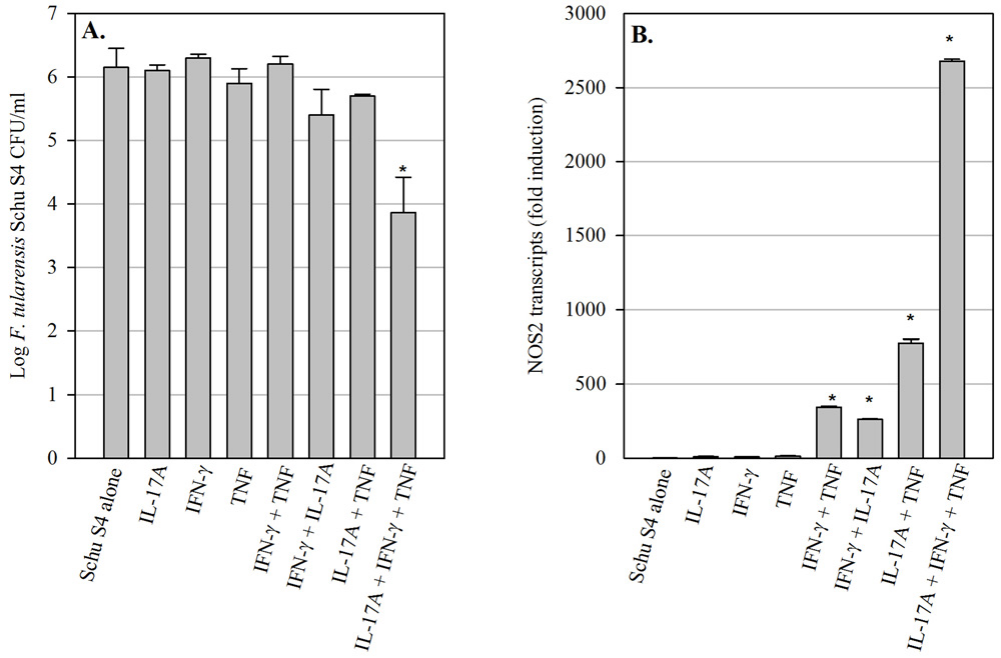
*F*. *tularensis* subspecies *tularensis* Schu S4-infected pulmonary epithelial cells are relatively resistant to the antimicrobial effects of cytokine treatment. (A) TC-1 lung epithelial cells were infected with Schu S4, and intracellular bacteria were enumerated after 72 h. Cultures were treated with recombinant cytokines immediately after infection as indicated, or untreated (‘Schu S4 alone’). (B) Changes in NOS2 gene expression in TC-1 cell cultures infected with Schu S4 and treated with recombinant cytokines. Fold induction values are expressed relative to ‘Schu S4 alone’ cultures. * = significantly different from cultures treated only with individual cytokines (P<0.05). Data are expressed as mean ± SD of triplicate cultures, and plots are representative of two experiments of similar design.

**Fig 3:**
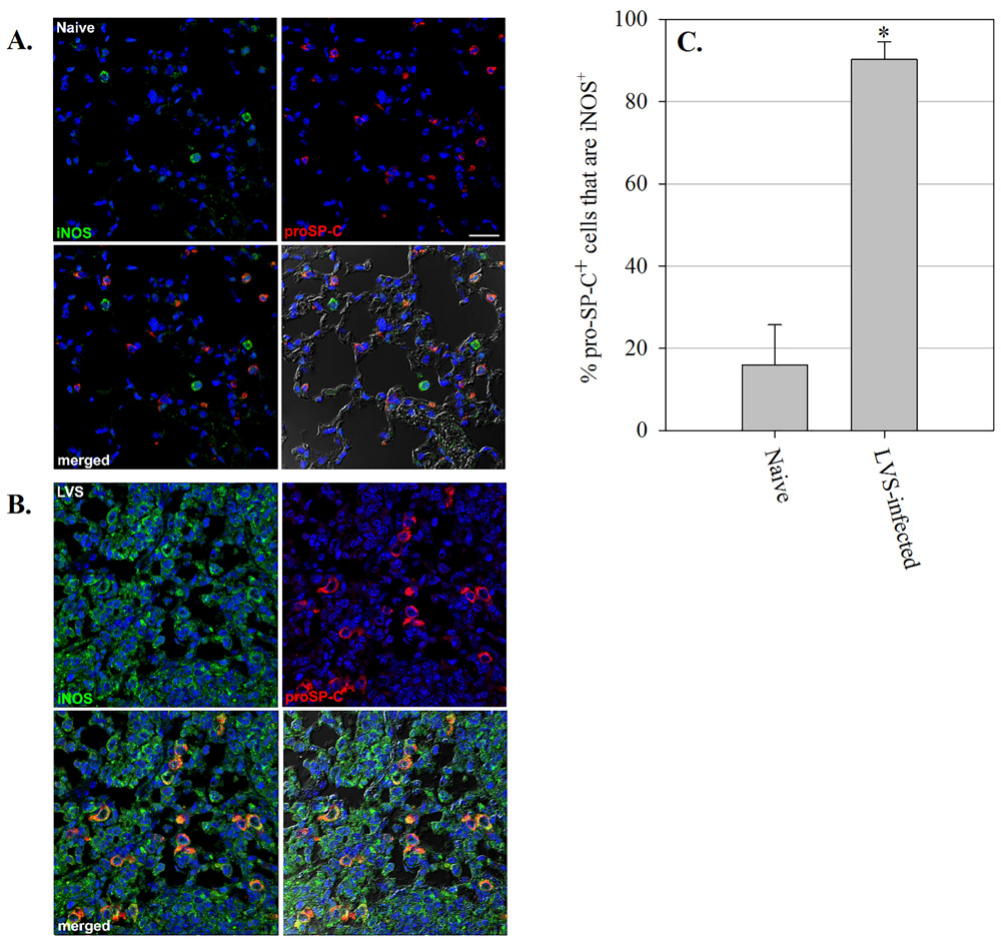
ATII cells in the lungs of mice produce iNOS during *in vivo* pulmonary *F*. *tularensis* LVS infection. (A) Naïve control mice, and (B) mice intranasally inoculated with 2x10^2^ CFU of *F*. *tularensis* LVS (lungs collected on day 7 after infection). Lungs were harvested and prepared for immunofluorescence analysis. (C) Percentage of pro-SP-C^+^ cells that stained positively for iNOS in naïve and LVS-infected mouse lungs. Five separate images were quantitated per mouse. Data are expressed as mean ± SD of individual mice. Nuclei were stained with Hoechst 33258 (blue) to visualize lung cells. Sections were probed with fluorescently labeled antibody to pro-SP-C (red) and iNOS (green) to identify lung epithelial cells expressing iNOS. The lower right corner of each panel is a DIC/merged image. Scale bar, 250 μm. Representative images are shown. * = *P*<0.001 as compared to naïve mice.

All experiments were repeated at least two to three times to assess reproducibility. Statisical analyses were performed on each experiment, and one representative experiment is shown for publication. All data were tested for equal variance and normal distribution (Shapiro-Wilk) prior to statistical testing. Following confirmation of equal variance and normal distribution, data were analyzed via one-way ANOVA followed by the Student-Newman-Keuls multiple stepwise comparison (for experiments with >2 experimental groups; Figs 1 and 2). For experiments with only two experimental groups (Fig 3), data were analyzed using a two-tailed Student’s *t*-test; *P*-values of 0.05 or less were considered significant.

## Results

### Cytokine-induced control of *F*. *tularensis* LVS growth in pulmonary epithelial cells is partially dependent upon RNI

Previous studies have shown that *F*. *tularensis* LVS invades and replicates in several lung epithelial cell lines, including the murine lung epithelial cell line TC-1, the human lung ATII cell line A459, and the mouse ATII-derived cell line MLE-12 [12, 16]. Of the three aforementioned cell lines, TC-1 cells exhibited the highest LVS invasion frequency [12], and have been used to define the intracellular trafficking of LVS during infection of pulmonary epithelial cells [16]. Since TC-1 cells are the best characterized pulmonary epithelial cell line with respect to *Francisella* infection, we used TC-1 cells to examine the impact of cytokines on control of LVS intracellular growth in pulmonary epithelial cells.

We first chose to focus our efforts on cytokines known to be essential for survival of primary murine LVS respiratory infection: IL-17A, IFN-γ, and TNF. Since all three of these cytokines are produced by both innate and adaptive immune cells in the lungs, they have the potential to influence pulmonary epithelial cell activities throughout LVS pulmonary infection. Recombinant cytokines were added to LVS-infected TC-1 cells immediately after infection, and growth of LVS in cytokine-treated and control cultures was evaluated after 72 hours. As seen in Fig 1A, LVS growth in TC-1 cells was only marginally affected by addition of either IL-17A or TNF individually (not significant; P > 0.05). In contrast, cultures containing IFN-γ demonstrated significant LVS growth inhibition (0.6 log_10_ CFU; P = 0.02), while cultures containing a combination of IL-17A and IFN-γ exhibited an enhanced effect on LVS growth control that was significantly greater than cultures treated with either IFN-γ or IL-17A alone (2.4 log_10_ CFU as compared to untreated cultures; P = 0.003 as compared to IFN-γ alone). Similarly, the combination of IFN-γ and TNF significantly reduced LVS growth (2.1 log_10_ CFU as compared to untreated cultures; P = 0.003 as compared to IFN-γ alone). Further, the combination of IL17A and TNF reduced LVS growth to a significantly greater extent than cultures treated with either TNF or IL-17A alone (1.5 log_10_ CFU as compared to untreated cultures; P = 0.02 as compared to IL-17A alone). Finally, the triple combination of IFN-γ, TNF, and IL-17A yielded maximal LVS growth inhibition (3.5 log_10_ CFU as compared to untreated cultures; P = 0.02 as compared to the IFN-γ and IL-17A combination). Of note, addition of recombinant IL-22 to the LVS-infected TC-1 cultures, either alone or in combination with the other cytokines, had no significant effect on LVS growth in TC-1 cells (data not shown).

Macrophages are well known to up-regulate expression of the inducible nitric oxide synthase gene (NOS2) in response to IFN-γ, but the ability of pulmonary epithelial cells to express NOS2 and associated RNI has not been extensively investigated. Therefore we next considered the possibility that cytokine treatment may induce expression of NOS2 transcripts in LVS-infected pulmonary epithelial cells. To this end, TC-1 cells were infected with LVS and treated with recombinant cytokines as described above; RNA was harvested on day 2 after infection and NOS2 transcripts were evaluated by Taqman qRT-PCR. Although IFN-γ is well known to induce NOS2 gene expression in macrophages, IFN-γ treatment of LVS-infected TC-1 cells had no substantial effect on NOS2 gene expression (Fig 1B); similarly, no nitrite was detected in the supernatant of these cultures (Fig 1C). However, NOS2 expression was significantly up-regulated in LVS-infected TC-1 cells treated with the double cytokine combinations (IFN-γ + TNF, IFN-γ + IL-17A, and IL-17A + TNF) as compared to cultures containing the single cytokine treatments; correspondingly, low but significant levels of nitrite were detected in the supernatants of these cultures (Fig 1B and 1C). Consistent with the high level of LVS growth inhibition observed in the triple cytokine combination cultures (containing IFN-γ, IL-17A, and TNF; Fig 1A), these cultures also exhibited the highest level of NOS2 up-regulation and nitrite production as compared to the other cytokine-treated cultures (Fig 1B and 1C). Of note, NOS2 expression was undetectable in uninfected TC-1 cultures treated with the aforementioned cytokine combinations (data not shown).

We next sought to determine whether the observed up-regulation of NOS2 gene expression and RNI production was sufficient to control LVS growth in cytokine-treated pulmonary epithelial cells. To this end, the iNOS inhibitor N^G^-monomethyl L-arginine (NMMA) was added to LVS-infected TC-1 cultures treated with recombinant cytokines, and LVS growth was determined after 72 hours. As seen in Fig 1D, the presence of NMMA resulted in a small but significant reversal of LVS growth control in the IFN-γ + IL-17A cultures (approximately 0.7 log_10_, *P*<0.05) and IL-17A + TNF cultures (approximately 0.7 log_10_, *P*<0.05), but had no detectable effect on the IFN-γ + TNF combination; in contrast, addition of NMMA to TC-1 cells containing the triple cytokine combination resulted in a much larger 2.1 log_10_ reversal of LVS killing (*P* = 0.006). Importantly, although NMMA had a significant effect on LVS killing by three of the cytokine combinations, substantial and significant LVS growth control remained in some of the cytokine combinations despite the presence of NMMA. Indeed, control of LVS growth in the presence of IFN-γ + TNF appeared to be completely independent of iNOS and RNI. Overall, these results demonstrate that, similar to macrophages, pulmonary epithelial cells produced RNI in response to cytokines; however, a significant amount of cytokine-induced LVS growth inhibition in these cells was RNI-independent.

### Highly virulent *F*. *tularensis* Schu S4 is relatively resistant to the antimicrobial activities of cytokine treated pulmonary epithelial cells

The virulent *F*. *tularensis* subspecies *tularensis* (*F*.*t*. *tularensis*) has been classified as a Tier 1 select agent due to its low infectious dose and ability to cause a lethal infection in humans when acquired via aerosol [1]. *F*.*t*. *tularensis* possesses several mechanisms by which its high virulence may be attributed, including the capacity to quickly suppress host inflammatory responses. Since *F*.*t*. *tularensis* Schu S4 has been shown to infect ATII cells in mouse lungs following intranasal infection [3], we were interested in determining whether virulent *F*.*t*. *tularensis* Schu S4 growth in pulmonary epithelial cells could, similar to LVS, be controlled by cytokine treatment. TC-1 cells were infected with Schu S4 at an MOI of 1:1 and treated with recombinant cytokines as described above; bacterial growth in the TC-1 monolayers was determined after 72 hours. Similar to LVS, Schu S4 grew unrestricted in TC-1 cells over the 72 hour time period (Fig 2A). However, unlike LVS, the double cytokine combinations did not significantly limit Schu S4 growth; only the triple cytokine combination (IFN-γ, TNF, and IL-17A) significantly reduced Schu S4 growth in TC-1 cells (approximately 2 log_10_ CFU for Schu S4, as compared to 3.5 log_10_ CFU for LVS). Thus, *F*.*t*. *tularensis* Schu S4 is relatively resistant to the growth inhibitory effects of cytokine-treated pulmonary epithelial cells.

We were next interested in determining whether the observed resistance of Schu S4 to cytokine-induced growth inhibition by TC-1 cells occurred despite NOS2 gene expression. As seen in Fig 2B, NOS2 expression was significantly up-regulated in Schu S4-infected cells treated with all of the double cytokine combinations, and appeared to be synergistically up-regulated in the triple cytokine combination. The magnitude of NOS2 gene up-regulation was comparable between similarly treated LVS-infected and Schu S4-infected TC-1 cells, suggesting that the lack of Schu S4 growth inhibition in these cells is not due to impaired NOS2 gene expression. Overall, these data demonstrate that the resistance of Schu S4 to the growth inhibitory effects of cytokine-activated ATII cells occurs despite robust expression of NOS2.

### Pulmonary epithelial cells produce iNOS during *F*. *tularensis* LVS intranasal infection *in vivo*

To determine whether lung epithelial cells produce iNOS during pulmonary *F*. *tularensis* LVS infection *in vivo*, we harvested lungs from mice 7 days after sublethal LVS intranasal infection, and examined co-localization of iNOS with pro-Surfactant Protein-C (pro-SP-C), a protein shown to be specifically produced by ATII cells [17]. The lungs of naïve uninfected mice exhibited marginal iNOS staining, and little co-localization of iNOS with pro-SP-C-positive cells (15.95 ± 9.74%; Fig 3A and 3C). However, as seen in Fig 3B and 3C, numerous ATII cells positive for pro-SP-C in the lungs of mice 7 days after LVS intranasal infection were also strongly positive for expression of iNOS (90.25 ± 4.31%). Not surprisingly, we further observed numerous cells in the lungs of infected mice that were positive for iNOS, but negative for pro-SP-C. Collectively, these results show that ATII cells, in addition to other lung cell types that likely include macrophages, produce iNOS during *in vivo* LVS pulmonary infection, suggesting that pulmonary epithelial cells use RNI to control LVS growth *in vivo* as well as *in vitro*.

## Discussion

The virulence of *F*. *tularensis* has long been associated with its ability to grow in macrophages, although it recently has become evident that *Francisella* proliferates in a wide variety of different host cell types both *in vitro* and *in vivo*. Although it is well known that IFN-γ activation severely limits *F*. *tularensis* replication in macrophages, little is known about the requirements for killing of *F*. *tularensis* in the non-macrophage host cell types that support replication of this organism. Here we sought to address this question through the use of a murine pulmonary epithelial cell line that has been well characterized with respect to LVS intracellular localization and growth. Using this cell line, we found that murine pulmonary epithelial cells possess the ability to inhibit intracellular growth of *F*. *tularensis* following activation with combinations of the cytokines IFN-γ, TNF, and IL-17A. Cytokine treatment induced RNI production by murine pulmonary epithelial cells in sufficient quantities to limit LVS growth *in vitro*. We further found that pulmonary epithelial cells produced iNOS protein during LVS respiratory infection *in vivo*.

Although macrophages have frequently been shown to produce RNI, reports of pulmonary epithelial cell RNI production are less frequent. A number of human and rodent pulmonary epithelial cell types have been shown to express NOS2 and RNI under various conditions such as lung injury [18], RSV infection [19], or cytokine and LPS treatment [20], but relatively few studies have examined pulmonary epithelial cell expression of NOS2 in the context of bacterial infection and cytokine activation. Roy *et al*. demonstrated that the A549 human alveolar epithelial cell line produced RNI following *M*. *tuberculosis* infection and treatment with IFN-γ and TNF [21]. Although the observed reduction in mycobacterial CFUs correlated with cytokine treatment, the role of RNI in mycobacterial growth inhibition was not assessed. However, consistent with our own findings, IL-17A and IFN-γ synergistically induced NOS2 expression and mediated RNI-dependent control of *Chlamydia muridarum* growth in a murine pulmonary epithelial cell line *in vitro* [22]. Here we show for the first time that the cytokine-induced production of RNI by murine pulmonary epithelial cells is an important mechanism by which these cells inhibit *F*. *tularensis* LVS growth. The double cytokine combinations of IFN-γ + TNF, IFN-γ + IL-17A and IL-17A + TNF, as well as the triple cytokine combination of IFN-γ + TNF + IL-17A, resulted in significant up-regulation of NOS2 transcripts in LVS-infected pulmonary epithelial cells. We further found that the triple cytokine combination had a synergistic effect on pulmonary epithelial cell RNI production that correlated with increased control of LVS growth. Importantly, addition of the iNOS inhibitor NMMA significantly reversed control of LVS growth in most of these cultures, confirming the role of RNI in LVS killing in cytokine-activated pulmonary epithelial cells.

In contrast to LVS, Schu S4 was much less susceptible to the antimicrobial activities of cytokine-treated pulmonary epithelial cells, despite robust levels of NOS2 gene expression by these cells. Correspondingly, Schu S4 has previously been shown to be more resistant to RNI than LVS [11]. Several genes have been identified in *Francisella* that can mediate resistance to RNI and reactive oxygen intermediates (ROI) *in vitro*, including catalase (KatG), alkyl-hydroperoxide reductase (AhpC), glutathione reductase (GpX), and a DyP-type peroxidase (FTT0086) [11, 23]. Since ROI can combine with nitric oxide to form peroxynitrite, a key mediator of IFN-γ-induced killing of LVS in macrophages *in vitro* [10], enzymes that neutralize ROI likely also contribute to *Francisella* resistance to RNI. Interestingly, a Schu S4 AhpC mutant was highly susceptible to *in vitro* killing by SIN-1, a chemical compound that spontaneously generates peroxynitrite [23]. This mutant also exhibited impaired growth in organs during *in vivo* intradermal infection of mice, suggesting that AhpC contributes to Schu S4 virulence [23]. However, possible differences in AhpC expression or activity between Schu S4 and LVS have yet to be investigated, so it remains unknown whether this gene contributes to the heightened resistance of Schu S4 to RNI. Regardless of the mechanism, it appears that the quantities of RNI generated by the cytokine-treated pulmonary epithelial cells were, in contrast to LVS, largely insufficient to limit Schu S4 intracellular growth.

In many extracellular bacterial infections, IL-17A modulates neutrophil activity by inducing production of cytokines that promote neutrophil expansion and survival (G-CSF and GM-CSF) in addition to chemokines that induce neutrophil recruitment (CXC chemokines) [24–26]. Moreover, IL-17A promotes production of antimicrobial peptides that directly contribute to pathogen destruction [27]. In contrast, IL-17A appears to have a more complex role in defense against *Francisella* infection. The primary function for IL-17A in *F*. *tularensis* LVS infection is proposed to be induction of IL-12 and IFN-γ by dendritic cells and macrophages, thus driving the development of critical Th1 responses required for clearance of the pathogen [28]. The resulting IFN-γ production subsequently acts on macrophages to mediate LVS killing [28]. Interestingly, the data presented here showed that IL-17A worked synergistically with IFN-γ and TNF to induce control of *F*. *tularensis* growth in pulmonary epithelial cells. Indeed, IFN-γ and TNF, either alone or in combination, were not sufficient to induce maximal killing and RNI production by pulmonary epithelial cells, but instead these cells also required the presence of IL-17A. Thus IL-17A may have an additional role in defense against *F*. *tularensis* infection that involves direct activation of pulmonary epithelial cells to produce optimal levels of bactericidal RNI that aid in bacterial clearance.

Although our results demonstrated that pulmonary epithelial cells utilized RNI to control LVS intracellular growth, we further observed that a significant proportion of cytokine-induced LVS growth inhibition was RNI-independent. In particular, pulmonary epithelial cells treated with IFN-γ + TNF produced minor amounts of RNI, but exhibited potent control of LVS intracellular growth that was unaffected by treatment with NMMA. This suggests that other unidentified antimicrobial mechanisms contribute to control of *F*. *tularensis* LVS intracellular growth in pulmonary epithelial cells. Interestingly, previous studies have reported RNI-independent control of *F*. *tularensis* growth in alveolar macrophages and bone-marrow-derived macrophages [29, 30], although the alternative mechanisms responsible for this activity have yet to be identified. Potential RNI-independent mechanisms that may limit *F*. *tularensis* intracellular growth include anti-microbial peptides [31], depletion of tryptophan via the activity of indolamine-2,3-dioxygenase [32], and iron sequestration [33]. Future studies will investigate the contribution of these and other mechanisms to control of *F*. *tularensis* intracellular growth in pulmonary epithelial cells.

Overall, our data demonstrate that pulmonary epithelial cells require a combination of IFN-γ, TNF and IL-17A in order to exert maximal control of *F*. *tularensis* intracellular growth. In response to these cytokines, pulmonary epithelial cells up-regulate NOS2 and produce RNI that inhibit *Francisella* growth. The ability of these cells to express iNOS both *in vitro* and during *in vivo* respiratory LVS infection revealed that pulmonary epithelial cells actively contribute to the control of *Francisella* infection through the production of antimicrobial products. Further, the observation that IL-17A was necessary to elicit maximum iNOS activity and *Francisella* killing by these cells underscores the importance of Th17 responses in defense against *Francisella* infection.
